# Mental health of airline pilots in France: insights from an anonymous online survey

**DOI:** 10.3389/fpubh.2025.1514812

**Published:** 2025-05-23

**Authors:** Madeleine Percheron, Bernard Prouvost-Keller, Jonathan Allouche, Michel Benoit, Christian Pradier

**Affiliations:** ^1^Département de Santé Publique, Centre Hospitalier Universitaire de Nice, Nice, France; ^2^Université Côte d’Azur, CoBTeK Laboratory, CHU de Nice, France; ^3^Université Côte d’Azur, UR2CA, CHU de Nice, France

**Keywords:** depressive disorder, anxiety disorder (AD), mental health, alcohol consumption, airline pilots, aviation medicine, suicidal thought

## Abstract

**Background:**

The German wings crash highlighted the need to better understand the mental health of airline pilots. Airline pilots are exposed to psychosocial risks, and they may constitute a population at risk of developing anxiety and depressive disorders. However, mental health remains difficult to assess in this population due to the risk of being declared unfit to fly. Scientific studies on mental disorders in airline pilots are rare, and the results are heterogeneous. To date, no study has been conducted describing anxiety or depressive disorders among European airline pilots.

**Method:**

We conducted a descriptive cross-sectional study using an anonymous online self-questionnaire. Pilots were recruited from the National Union of Airline Pilots between September 1 and October 16, 2022.

**Findings:**

Out of the 1,220 pilots surveyed, 25.4% of them suffered from anxiety according to the Hospital and Depression Scale (HAD): 14.4% suspected anxiety disorders and 11.0% confirmed anxiety disorders. Additionally, 13.1% of subjects reported depressive symptoms, including 8.9% suspected depressive disorders and 4.2% confirmed depressive disorders, according to the HAD. More than a third of the sample (40.1%) showed alcohol misuse.

**Interpretation:**

This study represents a major advancement in understanding the mental health of European airline pilots. This work highlights the need to implement prevention programs targeting profiles at risk of developing anxiety and/or depressive disorders. Our study also showed that a large proportion of subjects exhibited alcohol misuse, which requires prevention efforts to reduce health risks. In the future, conducting longitudinal studies would further strengthen our knowledge on this topic.

## 1 Introduction

### 1.1 Background

The crash of Germanwings Flight in March 2015 was caused by the suicide of the copilot. The cause of the accident was determined retrospectively by the experts of the Bureau d’Enquêtes et d’Analyses (BEA). This copilot had been suffering from severe depression for several weeks and had not informed the airline about it (1). This is not an isolated event. Other similar cases of suicides while in command have been described in aviation records, such as the crash of EgyptAir Flight 990 in 1999 and the crash of SilkAir Flight 185 in 1997 (2). The mental health of airline pilots is therefore a variable that can pose risks to aviation safety.

The International Civil Aviation Organization mandates that airline pilots undergo a specialized medical examination aimed at eliminating any somatic and/or psychiatric contra-indication to practice this profession. It is carried out in approved centres for aviation medicine. Aviation medical examiners (AME) are the only ones authorized to issue the medical fitness of airline pilots, or Class 1 medical certificate, which must be renewed every year (3).

According to the recommendations of the European Aviation Safety Agency (EASA), anxiety and depressive disorders are grounds for suspension of medical fitness (3).

Airline pilots are exposed to psychosocial risks and they may constitute a population at risk of developing anxiety and depressive disorders (4). However, mental health remains difficult to assess in this population due to the likelihood of being declared unfit by the AME (3). This leads to a under-reporting of psychological symptoms and the use of psychotropic treatments during medical fitness examinations (5–7).

Despite these concerns, research on the mental health of airline pilots remains limited, with heterogeneous results (8). According to the latest figures from 2018, there were 7,642 active airline pilots in France (9). The National Union of Airline Pilots (SNPL) represents the main pilot union in France, encompassing all airlines. Its membership rate reaches approximately 77% of employed pilots in France (10), which represents one of the largest national databases concerning them.

Existing studies highlight the role of occupational stress in pilots’ health outcomes (4), but large-scale data on anxiety and depressive disorders in European airline pilots are lacking.

### 1.2 Study objectives

Therefore, we aimed to describe the mental health of airline pilots by assessing anxiety and depressive dimensions, as well as the socio-demographic, professional factors, and potential comorbidities associated with them.

## 2 Materials and methods

### 2.1 Study design and participant recruitment

We conducted a descriptive cross-sectional study using an anonymous online self-survey. A link to the questionnaire was emailed to all SNPL members between September 1 and October 16, 2022, through the Drag’n Survey platform. The questionnaire was available in both French and English.

### 2.2 Questionnaire

The questionnaire included socio-demographic, professional, and medical data. Standardised and validated scales in both French and English were used to evaluate anxiety and depressive symptoms (Hospital Anxiety and Depression Scale, HAD-S) (11), personal alcohol consumption (Alcohol Use Disorder Identification Test-Concise) (12), and cannabis use (Cannabis Abuse Screening Test) (13).

#### 2.2.1 Hospital Anxiety and Depression Scale (HAD-S)

The HAD-S is an assessment scale for anxiety and depressive symptoms experienced in the past week. It consists of two subscales: one evaluating the depressive dimension and the other the anxious dimension. For each subscale, the interpretation is as follows: < 8: absence of symptoms, 8 to 10: suspected disorder, and > 10: confirmed disorder. This tool allows for the identification of anxiety symptoms with a sensitivity of 72% and a specificity of 96% (HAD-A). Similarly, it allows for the evaluation of depressive states with a sensitivity of 82% and a specificity of 92% (HAD-D) (11).

#### 2.2.2 Alcohol Use Disorder Identification Test-Concise (AUDIT-C)

The AUDIT-C is a validated scale for diagnosing alcohol misuse with a sensitivity of 86% and a specificity of 72%. It consists of 3 questions. A total score of 4 or higher for men and 3 for women suggests alcohol misuse. A score above 10 in both sexes should raise suspicion of dependence (12).

#### 2.2.3 Cannabis Abuse Screening Test (CAST)

The CAST is a tool for identifying cannabis misuse. Users are classified as low risk when they score below 3, as moderate risk when they score between 3 and 7, and as high risk for dependence when they score equal to or above 7 (13).

### 2.3 Outcome measures

The primary outcome measure was to determine anxiety and depression scores using the HAD-S scale.

The secondary outcome measure was to describe and measure the association of commonly found co-factors in anxiety and depressive disorders, including socio-demographic and occupational characteristics, health data, and alcohol and cannabis use.

### 2.4 Statistical analysis

We used JAMOVI software for data analysis. For continuous variables, t-test or Mann–Whitney test were chosen based on normality assumption. We used chi-square/Fisher’s exact tests for univariate analysis for anxiety or depression and the logistic regression model included participant numbers, univariate factors, and other relevant influences on anxiety or depression, maintaining a 5% alpha risk. Significance was defined as *p*-value <0.05.

## 3 Results

### 3.1 Participation description

We received 1,276 questionnaires, and 1,220 (95.6%) were included (56 responses were not considered in the analysis: 55 questionnaires reported an age equal to or older than 65 years, the age limit for practice, and 1 questionnaire was not usable).

### 3.2 Participant characteristics

#### 3.2.1 Socio-demographic characteristics

Out of the 1,220 participating pilots, 1,139 (93.3%) were of French nationality. We had 1,109 (90.9%) male participants, and the average age was 45.4 years. Among all respondents, 961 (78.8%) were in a relationship, and 868 (71.1%) had at least one child. Regarding the mention of financial difficulties, 180 (14.7%) reported experiencing them (Table 1).

**Table 1:** Socio-demographic characteristics.

		Subjects (*N* = 1,220)
Gender	Female	111 (9.1)
	Male	1,109 (90.9)
Age	Mean (standard-deviation or SD)	45.4 (9.8)
Nationality	France	1,139 (93.3)
	Europe (except France)	74 (6.1)
	Missing datas	7 (0.6)
Children	Yes	868 (71.1)
	No	335 (27.5)
	Missing datas	17 (1.4)
Financial difficulties	Yes	180 (14.7)
	No	907 (74.3)
	Missing datas	133 (11)
Marital status	In a relationship	961 (78.8)
	Single	242 (19.8)
	Missing datas	17 (1.4)

#### 3.2.2 Professional characteristics

Out of our 1,220 subjects, 626 (51.3%) held the position of captain. We had 743 (60.9%) pilots working for a national airline and 240 (19.7%) working for a low-cost airline. Among the entire sample, 505 (41.4%) worked in a city different from their place of residence. Regarding the type of operations, 551 (45.2%) crew members operated medium-haul flights, and 535 (43.9%) operated long-haul flights. In the studied population, 633 (51.9%) individuals worked between 40 and 70 h in the month preceding the questionnaire administration, and 635 (52.1%) were involved in multi-sector flights (Table 2).

**Table 2:** Professional characteristics.

		Subjects *N* = 1,220 (100%)
Status	Captain	626 (51.3)
	Copilot	571 (46.8)
	Missing data	23 (1.9)
Permanent contract	Yes	1,136 (93.1)
	No	47 (3.9)
	Missing data	37 (3)
Type of Arline	Legacy airline	743 (60.9)
	Low cost	240 (19.7)
	Corporate	44 (3.6)
	Cargo	43 (3.5)
	Other^a^	114 (9.3)
	Missing data	36 (3)
Working hours in the last thirty days	Not worked	56 (4.6)
	< 40 h	215 (17.6)
	Between 40 and 70 h	633 (51.9)
	> 70 h	264 (21.6)
	Missing data	52 (4.3)
Commuting^b^	Yes	505 (41.4)
	No	638 (52.3)
	Missing data	77 (6.3)
Permanent contract	Yes	1,136 (93.1)
	No	47 (3.9)
	Missing data	37 (3.0)
Type of operations		
Long haul flight^c^	Yes	535 (43.9)
	No	642 (52.6)
	Missing data	43 (3.5)
Medium haul flight^d^	Yes	551 (45.2)
	No	626 (51.3)
	Missing data	43 (3.5)
Regional flight^e^	Yes	256 (21.0)
	No	921 (75.5)
	Missing data	43 (3.5)
Number of operations	1	1,021 (83.7)
	> 1	156 (12.8)
	Missing datas	43 (3.5)
Flight types		
Night flight	Yes	649 (53.2)
	No	522 (42.8)
	Missing data	49 (4.0)
Jet lag >3 h	Yes	474 (38.9)
	No	697 (57.1)
	Missing data	49 (4.0)
Multi-sectors flight^f^	Yes	635 (52.1)
	No	536 (43.9)
	Missing data	49 (4.0)
Flight to the east	Yes	325 (26.7)
	No	846 (69.3)
	Missing data	49 (4.0)
Flight to the west	Yes	410 (33.6)
	No	761 (62.4)
	Missing data	49 (4.0)
Number of flight types	1 type of flight	543 (44.5)
	2 types of flight	203 (16.6)
	3 types of flight	109 (8.9)
	More than 3 types of flight	299 (24.5)

a Sector of activity excluding public transport.

b Place of work different from place of residence.

c Long haul flight >3500 km.

d Medium-haul flight: 1000–3500 km.

e Regional flight: 0–1000 km.

f Multi-sector flights characterized by multiple takeoffs and landings within a single workday and spanning several consecutive days.

#### 3.2.3 Medical data

Out of the 1,220 individuals surveyed, 388 (31.8%) had at least one medical history, and 117 (9.6%) had a psychiatric history. Excluding the use of sleeping pills, 316 pilots (25.9%) reported taking medication in the last 30 days. The use of medication for insomnia was reported by 102 (8.4%) participants, with 18 (17.6%) of them reporting taking it more than twice a week. Only 3 (0.2%) pilots reported having attempted suicide, and 33 (2.7%) experienced suicidal thoughts in the past year. The average fatigue level on the numeric scale was 5.7 out of 10.

More than a third of the participants, 497 (40.1%) pilots, scored indicating alcohol misuse according to the AUDIT-C scale. Among them, 8 (0.7%) were dependent. On the other hand, only 9 (0.8%) reported cannabis use in the past year.

We noted that 179 (14.7%) respondents sought external help for psychological reasons in the past year. A significant proportion had consulted a psychiatrist and/or a psychologist, accounting for 114 individuals (10.7%).

Among all 1,220 participants, 153 (12.5%) reported feeling isolated in dealing with their psychological distress, and 346 (28.3%) did not want to disclose their psychological symptoms to their AME for fear of losing their Class I medical certificate. However, 659 (54.0%) were willing to report their symptoms if a procedure ensured no impact on their career (Table 3).

**Table 3:** Medical datas.

		Subjects *N* = 1,220 (100%)
Medical history^a^	Yes	388 (31.8)
	No	832 (68.2)
Psychiatric history^b^	Yes	117 (9.6)
	No	1,103 (90.4)
Suicide attempt history	Yes	3 (0.2)
	No	1,066 (87.4)
	Missing data	151 (12.4)
Suicidal thoughts within 12 months	Yes	33 (2.7)
	No	1,016 (83.3)
	Missing data	171 (14.0)
Taking medication in the last 30 days	Yes	316 (25.9)
	No	803 (65.8)
	Missing data	101 (8.3)
Use of medication for insomnia in the last 30 days	Yes	102 (8.4)
	No	1,009 (82.7)
	Missing datas	109 (8.9)
Frequency of taking medication for insomnia	< Once per week	47 (46.1)
	1 to 2 times per week	35 (34.3)
	More than twice per week	18 (17.6)
	Missing data	2 (2.0)
AUDIT-C^c^	Safe use	524 (43.0)
	Misuse	489 (40.1)
	Dependence	8 (0.7)
	Missing data	199 (16.3)
Cannabis consumption within 12 months	Yes	9 (0.8)
	No	1,063 (87.1)
	Missing data	148 (12.1)
Numeric Scale Fatigue	Mean (SD)	5.7/10 (2.3)
	Missing data	132 (10.8)
Request for help^d^ for psychological reasons during the year	Yes	179 (14.7)
	No	887 (72.7)
	Missing datas	154 (12.6)
Types of helps requested for psychological reasons during the year (many possible responses)	Occupational physician and/or Aeromedical Examiner (AME)	21 (2.0)
	General practitioner	71 (6.7)
	Psychiatrist and / or psychologist	114 (10.7)
	Website or online forums	8 (0.8)
Number of different types of aid requested during the year for psychological reasons	1	129 (12.1)
	>2	39 (3.7)
	Missing data	154
Failure to report^e^ symptoms for fear of loss of Classe I certificate	Yes	346 (28.3)
	No	626 (51.3)
	Missing data	248 (20.4)
Declaration^e^ of symptoms if no impact on fitness to fly	Yes	659 (54.0)
	No	188 (15.4)
	Missing data	373 (30.6)
Feeling of isolation	Yes	153 (12.5)
	No	824 (67.5)
	Missing data	243 (20.0)

Categorical variables are presented by *N* (%) and compared using Chi-Squared test and continuous variables by mean (SD) and compared using one-way ANOVA. The *p*-value is significant at *p* < 0.05.

a Physical and/or psychiatric condition, self-reported and/or treated.

b Self-reported and/or treated psychiatric condition.

c AUDIT-C: Alcohol Use Disorders Identification Test (short version).

d Includes occupational physician and/or aeromedical examiner or AME, general practitioner, psychiatrist and/or psychologist, website and/or online forum.

e To the AME during the annual class I license renewal visit.

### 3.3 Comparative analysis of HAD-A and HAD-D profiles

#### 3.3.1 Anxiety disorders (HAD-A)

In our study, 310 participants (25.4%) had an abnormal score on the HAD-A (HAD-A ≥ 8). Among them, 176 (14.4%) were identified with a score between 8 and 10 (suspected anxiety disorder) and 134 (11.0%) with a score higher than 10 (confirmed anxiety disorder) (Table 4).

**Table 4:** Hospital anxiety and depression scale (HAD).

Sub-scale	Interpretation	Subjects *N* = 1,220 (100%)
HAD-A	No symptoms (score < 8)	778 (63.8)
	Suspected anxiety disorder (score between 8 and 10)	176 (14.4)
	Confirmed anxiety disorder (score > 10)	134 (11.0)
	Missing data	132 (10.8)
HAD-D	No symptoms (score < 8)	932 (76.4)
	Suspected depressive disorder (score between 8 and 10)	108 (8.9)
	Confirmed depressive disorder (score > 10)	51 (4.2)
	Missing data	129 (10.5)

In bivariate analysis, presented in Table 5, we observed a statistically significant association between different classes of HAD-A and sex (*p* = 0.025), marital status (*p* = 0.019), type of company (*p* = 0.002), hours worked in the last thirty days (*p* < 0.001), presence of financial difficulties (*p* < 0.001), presence of at least one medical history (*p* < 0.001), presence of psychiatric history (*p* < 0.001), use of insomnia medication (*p* < 0.001) and its frequency (*p* < 0.001), AUDIT-C score (*p* = 0.005), seeking help for psychological reasons during the year (*p* < 0.001), and feelings of isolation (*p* < 0.001). Additionally, there was a concurrent increase in self-reported fatigue scores on the numerical scale with the HAD-A score (*p* < 0.001).

**Table 5:** Main results of the comparison of respondent profiles according to the HAD-A.

		HAD-A			
Variable		No symptoms (score < 8) *N* = 778 (71.5%)	Suspected anxiety disorder (score between 8 and 10) *N* = 176 (16.2%)	Confirmed anxiety disorder (score ≥ 11) *N* = 134 (12.3%)	*P*
Gender	Female	60 (62.5)	16 (16.7)	20 (20.8)	0.025
	Male	718 (72.4)	160 (16.1)	114 (11.5)	
Age	Mean (SD)	45.8 (9.6)	44.5 (9.7)	44.8 (9.3)	0.157
Nationality	France	732 (71.1)	166 (16.3)	123 (12.0)	0.541
	Europe (Except France)	45 (68.2)	10 (15.2)	11 (16.7)	
Marital status	Single	145 (65.3)	38 (17.1)	39 (17.6)	0.019
	In a relationship	633 (73.3)	136 (15.7)	95 (11)	
Type of airline	Corporate	24 (54.5)	10 (22.7)	10 (22.7)	0.002
	Cargo	25 (64.1)	8 (20.5)	6 (15.4)	
	Legacy	523 (75.7)	98 (14.2)	70 (10.1)	
	Low-Cost	150 (68.5)	35 (16.0)	34 (15.5)	
	Autre^a^	56 (58.9)	25 (26.3)	14(14.7)	
Working hours in the last thirty days	Not worked	23 (44.2)	12 (23.1)	17 (32.7)	<0.001
	<40 h	138 (70.8)	32 (16.4)	25 (12.8)	
	Between 40 et 70 h	447 (75.0)	95 (15.9)	54 (9.1)	
	>70 h	170 (69.4)	37 (15.1)	38 (15.5)	
Financial difficulties	Yes	84 (48.3)	37 (21.3)	53 (30.5)	<0.001
	No	656 (76.8)	122 (14.3)	76 (8.9)	
Medical history^b^	Yes	233 (61.8)	61 (16.2)	83 (22.0)	<0.001
	No	545 (76.7)	115 (16.2)	51 (7.2)	
Psychiatric history^c^	Yes	51 (44.7)	25 (21.9)	38 (33.3)	<0.001
	No	727 (74.6)	151 (15.5)	96 (9.9)	
Suicidal thoughts within 12 months	Yes	8 (24.2)	7 (21.2)	18 (54.5)	<0.001
	No	750 (74.0)	160 (15.8)	103 (10.2)	
Use of regular medication	Yes	199 (66.6)	48 (16.1)	52 (17.4)	0.004
	No	572 (74.0)	123 (15.9)	78 (10.1)	
Use of medication for insomnia	Yes	48 (48.0)	23 (23.0)	29 (29.0)	<0.001
	No	725 (74.0)	152 (15.5)	103 (10.5)	
Frequency of taking medication for insomnia	< Once per week	27 (58.7)	12 (26.1)	7 (15.2)	<0.001
	1 to 2 times per week	17 (50.0)	9 (26.5)	8 (23.5)	
	More than twice per week	4 (22.2)	1 (5.6)	13 (72.2)	
HAD-D	No symptoms	738 (79.4)	137 (14.7)	55 (5.9)	<0.001
	Suspected disorder	33 (30.8)	31 (29.0)	43 (70.6)	
	Confirmed disorder	7 (13.7)	8 (15.7)	36 (70.6)	
AUDIT-C	Safe use	391 (74.8)	70 (13.4)	62 (11.9)	0.005
	Misuse	344 (70.5)	87 (17.8)	57 (11.7)	
	Dependence	3 (37.5)	1 (12.5)	4 (50.0)	
Cannabis consumption within 12 months	Yes	4 (44.4)	3 (33.3)	2 (22.2)	0.186
	No	763 (71.9)	169 (15.9)	129 (12.2)	
Numeric Scale Fatigue	Mean (SD)	5.2 (2.2)	6.7 (1.9)	7.5 (1.7)	<0.001
Request for help^d^ for psychological reasons during the year	Oui	70 (39.1)	55 (30.7)	54 (30.2)	<0.001
	Non	693 (78.3)	116 (13.1)	76 (8.6)	
Failure to report^e^ symptoms for fear of loss of Class I certificate	Oui	184 (53.3)	78 (22.6)	83 (24.1)	<0.001
	Non	509 (81.4)	77 (13.3)	39 (6.2)	
Declaration^e^ of symptoms if no impact on fitness to fly	Oui	438 (66.7)	121 (18.4)	98 (14.9)	0.007
	Non	147 (78.2)	26 (17.4)	15 (13.4)	
Feeling of isolation	Oui	48 (31.4)	37 (24.2)	68 (44.4)	<0.001
	Non	651 (79.2)	122 (14.8)	49 (6.0)	

HAD-A: Hospital Anxiety and Depression Scale: Score for Anxiety, HAD-D: Hospital Anxiety and Depression Scale: Score for Depression. AUDIT-C: Alcohol Use Disorders Identification Test (short version), Categorical variables are presented by *N* (%) and compared using Chi-Squared Test and continuous variables by mean (SD) and compared using One-Way ANOVA. The *p*-value is significant at *p* < 0.05.

a Sector of activity excluding public transport.

b Physical and/or psychiatric condition, self-reported and/or treated.

c Self-reported and/or treated psychiatric condition.

d Includes occupational physician and/or Aeromedical examiner or AME, general practitioner, psychiatrist and/or psychologist, website and/or online forum.

e To the AME during the annual Class I license renewal visit.

After including variables in a multivariate logistic regression model (Table 6), several factors remained significantly associated with the occurrence of an HAD-A score > 10 (confirmed anxiety disorder) compared to an HAD-A score < 8 (absence of symptoms). These factors included female gender (OR: 4.54 [2.00–10.35], *p* < 0.001), long-term medication use (OR: 2.82 [1.58–5.05], *p* < 0.001), feelings of isolation (OR: 4.49 [2.36–8.52], *p* < 0.001), engagement in multi-sector flights (OR: 1.81 [1.00–3.27], *p* = 0.05), abnormal HAD-D score (OR: 1.54 [1.38–1.72], *p* < 0.001), and fatigue score (OR: 1.44 [1.21–1.72], *p* < 0.001). However, working more than 40 h seemed to protect against the occurrence of a confirmed anxiety disorder (OR: 0.43 [0.20–0.95], *p* = 0.037, for the 40-70-h time range, and OR: 0.39 [0.16–0.98], *p* = 0.045 for the >70-h range).

**Table 6:** HAD-A multivariate analysis.

	Variable	Comparison	Odds ratio [IC 95%]	*p*
Suspected anxiety disorder versus no symptoms	Gender	Female–Male	1.65 [0.80–3.43]	0.177
	Financial difficulties	Yes–No	1.50 [0.88–2.56]	0.141
	HAD-D	HAD_D	1.33 [1.22–1.45]	< 0.001
	Numeric Scale Fatigue	NS Fatigue	1.26 [1.12–1.42]	< 0.001
	Feeling of isolation	Yes–No	1.48 [0.82–2.68]	0.19
	Multi-sector flights	Yes–No	1.98 [1.27–3.06]	0.002
	Failure to report symptoms for fear of loss of Classe I certificate	Yes–No	1.97 [1.27–3.05]	0.002
	Working hours in the last thirty days	Not worked – < 40 h	2.58 [0.94–7.11]	0.066
		Between 40 h and 70 h – < 40 h	0.81 [0.45–1.47]	0.489
		> 70 h – < 40 h	0.64 [0.31–1.30]	0.217
	Use of regular medication	Oui–Non	1.02 [0.65–1.60]	0.939
Confirmed anxiety disorder versus no symptoms	Gender	Female–Male	4.54 [2.00–10.35]	< 0.001
	Financial difficulties	Yes-No	1.90 [0.97–3.71]	0.061
	HAD-D	HAD_D	1.54 [1.38–1.72]	< 0.001
	Numeric scale fatigue	NS Fatigue	1.44 [1.21–1.72]	< 0.001
	Feeling of isolation	Yes–No	4.49 [2.36–8.52]	< 0.001
	Multi-sector flights	Yes–No	1.81 [1.00–3.27]	0.05
	Non reporting symptoms to AME	Yes–No	2.67 [1.48–4.80]	0.001
	Working hours in the last thirty days	Not worked – < 40 h	2.49 [0.73–8.54]	0.146
		Between 40 h and 70 h – < 40 h	0.43 [0.20–0.95]	0.037
		> 70 h – < 40 h	0.39 [0.16–0.98]	0.045
	Use of regular medication	Yes–No	2.82 [1.58–5.05]	< 0.001

Multinomial logistic regression model. Comparison: on the left the compared level and on the right the reference level. HAD-A: Hospital Anxiety and Depression Scale: Score for Anxiety, HAD-D: Hospital Anxiety and Depression Scale: Score for Depression, EN: Numerical Scale. Significant *p* value < 0.05.

We also found several factors significantly associated with the occurrence of an HAD-A score between 8 and 10 (suspected anxiety disorder) compared to the absence of symptoms. These factors included engagement in multi-leg flights (OR: 1.98 [1.27–3.06], *p* = 0.002), abnormal HAD-D score (OR: 1.33 [1.22–1.45], *p* < 0.001), and fatigue (OR: 1.26 [1.12–1.42], *p* < 0.001).

#### 3.3.2 Depressive disorders (HAD-D)

In our sample, 159 (13.1%) individuals had an abnormal score on the HAD-D (HAD-D ≥ 8). Among them, 108 (8.9%) were identified with a score between 8 and 10 (suspected depressive symptoms) and 51 (4.2%) with a score > 10 (confirmed depressive symptoms) (Table 4).

In Table 7, the bivariate analysis revealed significant associations between various HAD-D categories and factors including nationality (*p* = 0.05), type of company (*p* = 0.017), working hours (*p* < 0.001), financial difficulties (*p* < 0.001), medical history (*p* < 0.001), psychiatric history (*p* < 0.001), use of insomnia medication and its frequency (*p* < 0.001, *p* = 0.001), AUDIT-C score (*p* = 0.02), and feelings of isolation (*p* < 0.001). Like HAD-A, there was a concurrent increase in self-rated fatigue scores on the numeric scale with rising HAD-D scores (*p* < 0.001).

**Table 7:** Main results of the comparison of respondent profiles according to the HAD-D.

		HAD-D			
Variable		No symptoms (score < 8) *N* = 932 (85.4%)	Suspected depressive disorder (score between 8 and 10) *N* = 108 (9.9%)	Confirmed depressive disorder (score > 10) *N* = 51 (4.7%)	*P*
Gender	Female	82 (85.4)	13 (13.5)	1 (1.0)	0.112
	Male	850 (85.4)	95 (9.5)	50 (5.0)	
Age	Mean (SD)	45.6 (9.7)	44.8 (9.2)	45.2 (9.0)	0.680
Nationality	France	881 (86.2)	94 (9.3)	46 (4.5)	0.005
	Europe (except France)	50 (72.7)	14 (19.7)	5 (7.6)	
Marital status	Single	189 (84.8)	23 (10.3)	11 (4.9)	0.953
	In a relationship	741 (85.6)	85 (9.8)	40 (4.6)	
Type of airline	Corporate	34 (77.3)	6 (13.6)	4 (9.1)	0.017
	Cargo	32 (80.0)	5 (12.5)	3 (7.5)	
	Legacy	615 (88.7)	56 (8.1)	22 (3.2)	
	Low-Cost	174 (79.5)	29 (12.2)	16 (7.3)	
	Other^a^	77 (88.1)	12 (12.6)	6 (6.3)	
Working hours in the last thirty days	Not worked	35 (67.3)	10 (19.2)	7 (13.7)	<0.001
	<40 h	172 (87.8)	20 (10.2)	4 (2.0)	
	Between 40 et 70 h	525 (87.8)	54 (9.0)	19 (3.2)	
	>70 h	200 (81.6)	24 (9.8)	21 (8.6)	
Financial difficulties	Yes	116 (66.7)	34 (19.5)	24 (13.8)	<0.001
	No	766 (89.4)	67 (7.8)	24 (2.8)	
Medical history^b^	Yes	291 (77.0)	60 (15.9)	27 (7.1)	<0.001
	No	641 (89.9)	48 (6.7)	24 (3.4)	
Psychiatric history^c^	Yes	72 (63.2)	25 (21.9)	17 (14.9)	<0.001
	No	860 (88.0)	83 (8.5)	34 (3.5)	
Use of regular medication	Yes	243 (81.0)	42 (14.0)	15 (5.0)	0.013
	No	677 (87.4)	63 (8.1)	35 (4.5)	
Use of medication for insomnia	Yes	68 (68.0)	22 (22.0)	10 (10.0)	<0.001
	No	858 (87.3)	86 (8.7)	39 (4.0)	
Frequency of taking medication for insomnia	< Once per week	37 (80.4)	6 (13.0)	3 (6.5)	0.001
	1 to 2 times per week	23 (67.6)	10 (29.4)	1 (2.9)	
	More than twice per week	7 (38.9)	5 (27.8)	6 (33.3)	
HAD-A	No symptoms	738 (94.9)	33 (4.2)	7 (0.9)	<0.001
	Suspected disorder	137 (77.8)	31 (17.6)	8 (4.5)	
	Confirmed disorder	55 (41.0)	43 (32.1)	36 (26.9)	
Numeric scale fatigue	Mean (SD)	5.5 (2.2)	7.1 (1.8)	8.0 (1.5)	<0.001
Suicidal thoughts within 12 months	Yes	12 (36.4)	9 (27.3)	12 (36.4)	<0.001
	No	892 (87.8)	90 (8.9)	34 (3.3)	
AUDIT-C	Safe use	462 (88.3)	42 (8.0)	20 (3.8)	0.020
	Misuse	411 (84.0)	54 (11.0)	24 (4.9)	
	Dependence	5 (62.5)	1 (12.5)	2 (25.0)	
Cannabis consumption within 12 months	Yes	7 (77.8)	2 (22.2)	0 (0.0)	0.396
	No	907 (85.3)	105 (9.9)	51 (4.8)	
Request for help^d^ for psychological reasons during the year	Yes	115 (64.2)	47 (26.3)	17 (9.5)	<0.001
	No	794 (89.5)	59 (6.7)	34 (3.8)	
Feeling of isolation	Yes	83 (54.2)	42 (27.5)	28 (18.3)	<0.001
	No	752 (91.3)	54 (6.6)	18 (2.2)	
Failure to report^e^ symptoms for fear of loss of Class I certificate	Yes	250 (72.3)	58 (16.8)	38 (11.0)	<0.001
	No	576 (92.0)	39 (6.2)	11 (1.8)	
Declaration^e^ of symptoms if no impact on fitness to fly	Yes	545 (82.7)	77 (11.7)	37 (5.6)	0.319
	No	164 (87.2)	17 (9.0)	7 (3.7)	

HAD-A: Hospital Anxiety and Depression Scale: Score for Anxiety, HAD-D: Hospital Anxiety and Depression Scale: Score for Depression. AUDIT-C: Alcohol Use Disorders Identification Test (short version), categorical variables are presented by *N* (%) and compared using Chi-Squared test and continuous variables by mean (SD) and compared using one-way ANOVA. The *p*-value is significant at *p* < 0.05.

a Sector of activity excluding public transport.

b Physical and/or psychiatric condition, self-reported and/or treated.

c Self-reported and/or treated psychiatric condition.

d Includes occupational physician and/or aeromedical examiner or AME, general practitioner, psychiatrist and/or psychologist, website and/or online forum.

e To the AME during the annual class I license renewal visit.

After including the variables in a multivariate logistic regression model (Table 8), several factors remained significantly associated with the occurrence of an HAD-D score > 10 (confirmed depressive symptoms) compared to an HAD-D score < 8 (absence of symptoms). These factors included European nationality excluding France (odds ratio: 7.91 [2.15–31.79], *p* = 0.002), presence of suicidal ideation in the past year (OR: 4.40 [1.07–18.13], *p* = 0.04), presence of financial difficulties (OR: 3.41 [1.37–8.48], *p* = 0.008), fatigue (OR: 1.89 [1.35–2.63], *p* < 0.001), and abnormal HAD-A score (OR: 1.48 [1.28–1.71], *p* < 0.001). On the other hand, taking medication for insomnia (0.18 [0.033–0.98], *p* = 0.047) and consulting a general practitioner for psychological reasons (OR: 0.12 [0.02–0.66], *p* = 0.015) seemed to protect against confirmed depressive symptoms.

**Table 8:** HAD-D multivariate analysis.

	Variable	Comparison	Odds Ratio [IC 95%]	*p*
Suspected depressive disorder versus no symptoms	Consultation with a general practitioner (GP)	Yes – No	1.28 [0.50–3.27]	0.602
	Feeling of isolation	Yes – No	2.78 [1.49–5.17]	0.001
	HAD-A	HAD-A	1.24 [1.14–1.36]	< 0.001
	Numeric Scale Fatigue	NS Fatigue	1.24 [1.06–1.45]	0.008
	Financial difficulties	Yes – No	2.40 [1.29–4.48]	0.006
	Nationality	EU – FR	4.90 [2.10–11.40]	< 0.001
	Single	Yes – No	0.46 [0.23–0.95]	0.037
	Use of regular medication	Yes – No	3.26 [1.04–10.20]	0.042
	Use of medication for insomnia	Yes – No	0.40 [0.12–1.26]	0.116
	Suicidal thoughts within 12 months	Yes – No	2.05 [0.62–6.73]	0.237
	No help requested for psychological reasons	Yes – No	0.38 [0.18–0.82]	0.013
Confirmed depressive disorder versus no symptoms	Consultation with a GP	Yes – No	0.12 [0.02–0.66]	0.015
	Feeling of isolation	Yes – No	3.68 [1.42–9.52]	0.007
	HAD-A	HAD- A	1.48 [1.28–1.71]	< 0.001
	Numeric Scale Fatigue	NS Fatigue	1.89 [1.35–2.63]	< 0.001
	Financial difficulties	Yes – No	3.41 [1.37–8.48]	0.008
	Nationality	UE – FR	7.91 [2.15–31.79]	0.002
	Single	Yes – No	0.35 [0.11–1.08]	0.068
	Use of regular medication	Yes – No	4.11 [0.80–21.06]	0.09
	Use of medication for insomnia	Yes – No	0.18 [0.033–0.98]	0.047
	Suicidal thoughts within 12 months	Yes – No	4.40 [1.07–18.13]	0.04
	No help requested for psychological reasons	Yes – No	0.32 [0.11–0.92]	0.035

Multinomial logistic regression model. Comparison: on the left the compared level and on the right the reference level. HAD-A: Hospital Anxiety and Depression Scale: Score for Anxiety, HAD-D: Hospital Anxiety and Depression Scale: Score for Depression, EN: Echelle Numérique. *p* value significative < 0.05.

We also found several factors significantly associated with an HAD-D score between 8 and 10 (suspected depressive symptoms) compared to the absence of symptoms. These factors included nationality (OR: 4.90 [2.10–11.40], *p* < 0.001), long-term medication use (OR: 3.26 [1.04–10.20], *p* = 0.042), presence of financial difficulties (OR: 2.40 [1.29–4.48], *p* = 0.006), fatigue (1.24 [1.06–1.45], *p* = 0.008), and abnormal HAD-A score (1.24 [1.14–1.36], *p* < 0.001). On the other hand, being unmarried seemed to protect against the occurrence of suspected depressive symptoms (OR: 0.46 [0.23–0.95], *p* = 0.037).

## 4 Discussion

To date, this work is the first descriptive study focusing on the mental health of airline pilots in Europe. With its 1,220 participants, it provides a significant sample describing anxiety and depressive disorders within this population.

Our findings regarding depressive disorders compare closely to those of the largest investigation conducted on this subject in 2016 by Wu et al. This observational cross-sectional study using an anonymous self-survey showed that 233 (12.6%) out of the 1848 airline pilots surveyed had depressive symptoms (8). The authors used the PHQ-9 (Patient Health Questionnaire-9) as a screening instrument for depression with a threshold of 10, which is comparable to the threshold of 8 used in the HAD-S (14). A New Zealand study on the health of technical flight crew showed that out of 856 airline pilots surveyed, only 11 (1.9%) reported suffering from depression and 6 (1%) from anxiety. However, this study was not anonymous, and the authors acknowledged that the prevalence of anxiety and depression were likely underestimated due to the risk of flight disqualification (15).

According to the latest survey by Public Health France in September 2022, during the period of our study, among individuals in the higher socioeconomic category, 15.2% of respondents reported a score > 10 on the HAD-D scale. Regarding anxiety, 22.4% had an HAD-A score > 10 (16). The score of confirmed anxiety or depressive disorders in our sample of airline pilots is therefore lower than that found in the French population.

Among the surveyed pilots, 33 (2.7%) reported suicidal thoughts in the past year. In the scientific literature, there is very little data on the prevalence of suicidal thoughts among airline pilots. An analysis of airplane accident reports in the United States from 1993 to 2012 estimated that out of 7,244 fatal accidents, 24 (0.33%) were caused by pilot suicide (17). However, this study only considered completed suicides and not the prevalence of suicidal thoughts. The most recent data on suicidal thoughts among airline pilots comes from the study by Wu et al. (8). They showed that the prevalence of suicidal thoughts was 4.1% in their sample, which is comparable to the rate found in our population.

Among the socio-professional characteristics, we found that multi-sector flights increase the risk of confirmed anxiety disorders (HAD-A > 10) by nearly 2. Multi-sector flights, involve multiple trips within the same working day. This type of flight is demanding due to time pressure and the repetition of takeoff and landing phases, where the pilot’s cognitive load is highest (18). The work by Feijó et al. (19) showed that workload increased the risk of developing common mental disorders (CMD), including anxiety.

One of the most concerning findings of our study is the high prevalence of alcohol misuse among airline pilots. More than one-third of the sample (489 pilots or 40.1%) had an AUDIT-C score indicating risky alcohol use, and 8 (0.7%) met the criteria for alcohol dependence. Notably, 57% of pilots with confirmed depressive disorders (HAD-D > 10) reported alcohol misuse. These findings align with previous research indicating a strong association between alcohol consumption and mental health disorders. According to the French Society of Addiction Medicine, over one-third of individuals with alcohol misuse will experience a psychiatric comorbidity during their lifetime, particularly depression and anxiety disorders (20). There is a continuum between these different forms of use.

Our study shows a correlation between HAD-S scores and alcohol misuse in bivariate analysis. The links between mental health and alcohol have been extensively described in the scientific literature. According to the French Society of Addiction Medicine, over one-third of individuals with alcohol misuse will experience a psychiatric comorbidity during their lifetime, particularly depression and anxiety disorders (20). The anxiolytic properties of alcohol make it a commonly used substance among individuals with anxiety and depressive disorders, but chronic consumption exacerbates psychiatric symptoms over time (21). Some countries, such as Japan, already mandate pre-flight breathalyzer tests for pilots (22). Introducing such measures across more jurisdictions could help mitigate immediate risks related to acute alcohol consumption. Additionally, for chronic alcohol use, biological markers such as carbohydrate-deficient transferrin (CDT), mean corpuscular volume (MCV) and the gamma glutamyl transferase (GGT) markers could be implemented for systematic annual screening. These markers are a well-established biomarker for chronic alcohol consumption (23). Establishing standardized guidelines for marker normalization before a pilot is deemed fit to fly could enhance safety while promoting rehabilitation.

On the other hand, only 9 (0.8%) participants reported using cannabis in the past year. The low number of users could be explained by the implementation of random drug screening following the Germanwings accident in 2015 (4) and the slow elimination of tetrahydrocannabinol (THC), the psychoactive substance in cannabis (24).

We showed that the self-rated average fatigue on a numerical scale was 5.7/10. Several reasons can explain the observed links between fatigue and anxiety-depressive disorders in our study. “Jet lag” is common among pilots. Its manifestations include fatigue, irritability, and sometimes moderate depression (25). Working irregular hours increases the risk of depressive pathology (26). Finally, fatigue is one of the criteria for a major depressive episode according to the DSM-V (27).

In our study, 102 (8.4%) subjects reported taking medication for insomnia. Among them, 47 (46.1%) took it less than once a week, 35 (34.3%) took it 1 to 2 times a week, and 18 (17.6%) took it more than twice a week. Due to their profession, pilots are prone to insomnia. The links between insomnia and depression are complex. Insomnia is both a symptom and a risk factor for the onset of depression (28). Insomnia is included in the diagnostic criteria for a major depressive episode in the DSM-V (27). Conversely, fragmented sleep is accompanied by a stress reaction with hypothalamo-pituitary hyperactivation and carries a high risk of depression (28). Our study showed that taking insomnia medication more than twice a week was protective against a score of HAD-D > 10 (confirmed depressive disorder) (OR: 0.18).

However, the treatment of sleep disorders in airline pilots, should be approached with caution. Certain medication classes, such as benzodiazepine-type hypnotics, can affect cognitive performance and cause sedation. With chronic use, dependence is even possible. The same applies to sedating antihistamines, one of which is available over the counter in France, which can also cause sedation and cognitive impairments (29). Non-pharmacological strategies could be of interest in this population, such as cognitive-behavioral therapy, which has shown its effectiveness in chronic insomnia (29). It is also important to note that the prevention of insomnia and fatigue in airline pilots should consider the risk factors associated with their work. Prevention strategies can be implemented. Further studies on the subject would be interesting to determine the characteristics of insomnia in this population and its contributing factors.

Being of foreign nationality increased the risk of developing a confirmed depressive disorder (score of HAD-D > 10) by almost 8 times. Several studies conducted in populations of expatriate workers have shown that they are at risk of developing anxiety and/or depression-related mental disorders (30).

The risk of unfitness for duty acts as a barrier to reporting psychological symptoms to AME (8, 19). Our study showed that 659 (54.0%) pilots would be willing to report these symptoms provided that a procedure guarantees no impact on their career. As suggested by the BEA is in its report on the Germanwings crash (1), the self-declaration of anxiety and depressive symptoms to AME could be strengthened by mitigating the socio-economic risks associated with flight unfitness.

In close comparison to the study by Wu et al. (8), limitations of this work include a potential underestimation of the score of anxiety and/or depression due to a possible lower participation rate among individuals with more severe disorders compared to those without. This may have led to an underestimation of the true score of depression and anxiety during the survey period. Conversely, an upward bias may have occurred if individuals with these disorders were more likely to participate in the survey than those without symptomatology due to their familiarity with the study’s objective (8).

Another limitation of our study was the lack of statistical representativeness of our sample compared to the target population. We found a significant difference in terms of gender and job function. However, there was no significant difference in terms of the proportion of fixed-term contracts (CDD) and age. This lack of representativeness makes the results non-generalisable to the entire population of pilots affiliated with SNPL or even to the general population of airline pilots.

Although the HAD-S is a validated scale for screening anxiety and depression, it would be useful to propose medical interviews to confirm the diagnoses of anxiety and depressive disorders.

## 5 Conclusion

This study represents a major advancement in understanding the mental health of European airline pilots. Despite anxiety and depression levels are lower than those observed in higher socioeconomic categories in France, the results indicate that a significant proportion of pilots suffer from psychological symptoms. This work highlights the need to implement prevention programs targeting profiles at risk of developing anxiety and/or depressive disorders. Our study also showed that a large proportion of subjects exhibited alcohol misuse, which requires prevention efforts to reduce health risks. However, it is crucial to first establish measures aimed at mitigating the socio-economic risks associated with the loss of a license for medical reasons to encourage the self-reporting of anxiety and/or depressive symptoms to the AME. In the future, conducting longitudinal studies would be relevant to further strengthen our knowledge on this topic, which is rarely addressed in the scientific literature.

## Data Availability

The raw data supporting the conclusions of this article will be made available by the authors, without undue reservation.

## References

[1] Bureau d’Enquêtes et d’Analyse. Final report–Germanwings Airbus A320-211 (D-AIPX) accident 24 march 2015–BEA2015-0125.En. (2016). Available online at: https://www.bea.aero/uploads/tx_elydbrapports/BEA2015-0125.en-LR.pdf

[2] National Transportation Safety Committee. Aircraft accident report SilkAir flight MI 185. Palembang: Department of Communications Republic of Indonesia (1980).

[3] European Union Aviation Safety Agency. Easy Access Rules for Medical Requirements [Internet] (2020). Available online at: https://www.easa.europa.eu/sites/default/files/dfu/Easy_Access_Rules_for_Medical_Requirements.pdf

[4] Cullen P Cahill J Gaynor K. A qualitative study exploring well-being and the potential impact of work-related stress among commercial airline pilots. Aviat Psychol Appl Hum Fact. (2021) 11:1–12. doi: 10.1027/2192-0923/a000199

[5] Hoffman WR Patel PK Aden J Willis A Acker JP Bjerke E. Multinational comparison study of aircraft pilot healthcare avoidance behaviour. Occup Med. (2023) 73:434–8. doi: 10.1093/occmed/kqad091, PMID: 37658781

[6] Sen A Akin A Canfield DV. Chaturvedi AK. Medical histories of 61 aviation accident pilots with postmortem SSRI antidepressant residues. ASEM. (2007) 78:1055–9. doi: 10.3357/ASEM.2030.2007 18018438

[7] Cross DS Wallace R Cross J Coimbra Mendonca F. Understanding pilots’ perceptions of mental health issues: a qualitative phenomenological investigation among airline pilots in the United States. Cureus. (2024). Available online at: https://www.cureus.com/articles/276591-understanding-pilots-perceptions-of-mental-health-issues-a-qualitative-phenomenological-investigation-among-airline-pilots-in-the-united-states 10.7759/cureus.66277 PMC11302551 39108765

[8] Wu AC Donnelly-McLay D Weisskopf MG McNeely E Betancourt TS Allen JG. Airplane pilot mental health and suicidal thoughts: a cross-sectional descriptive study via anonymous web-based survey. Environ Health. (2016) 15:121. doi: 10.1186/s12940-016-0200-6, PMID: 27974043 PMC5157081

[9] Caisse de Retraite du Personnel Navigant. Chiffres clés. (2018). Available online at: https://www.crpn.fr/la-crpn/chiffres-cles/

[10] SNPL. Available online at: https://snpl.com

[11] Bocéréan C Dupret E. A validation study of the hospital anxiety and depression scale (HADS) in a large sample of French employees. BMC Psychiatry. (2014) 14:354. doi: 10.1186/s12888-014-0354-0, PMID: 25511175 PMC4476305

[12] Gache P Michaud P Landry U Accietto C Arfaoui S Wenger O. The alcohol use disorders identification test (AUDIT) as a screening tool for excessive drinking in primary care: reliability and validity of a French version: alcoholism. Clin Exp Res. (2005) 29:2001–7. doi: 10.1097/01.alc.0000187034.58955.64, PMID: 16340457

[13] Observatoire Français des Drogues et des Tendances addictives. Détection des usages problématiques de cannabis: le Cannabis Abuse Screening Test (CAST). (2013). Available online at: https://www.ofdt.fr/publications/collections/notes/detection-des-usages-problematiques-de-cannabis-le-cannabis-abuse-screening-test-cast

[14] Hansson M Chotai J Nordstöm A Bodlund O. Comparison of two self-rating scales to detect depression: HADS and PHQ-9. Br J Gen Pract. (2009) 59:e283–8. doi: 10.3399/bjgp09X454070, PMID: 19761655 PMC2734374

[15] Sykes AJ Larsen PD Griffiths RF Aldington S. A study of airline pilot morbidity. Aviat Space Environ Med. (2012) 83:1001–5. doi: 10.3357/ASEM.3380.2012, PMID: 23066624

[16] Santé Publique France. CoviPrev: une enquête pour suivre l’évolution des comportements et de la santé mentale pendant l’épidémie de COVID-19 [Internet]. Available online at: https://www.santepubliquefrance.fr/etudes-et-enquetes/coviprev-une-enquete-pour-suivre-l-evolution-des-comportements-et-de-la-sante-mentale-pendant-l-epidemie-de-covid-19#block-249162

[17] Vuorio A Laukkala T Navathe P Budowle B Eyre A Sajantila A. Aircraft-assisted pilot suicides: lessons to be learned. Aviat Space Environ Med. (2014) 85:841–6. doi: 10.3357/ASEM.4000.2014, PMID: 25199127

[18] François M Liévin D Mouzé-Amady M. Activité, charge de travail et stress du personnel navigant des compagnies aérienne: la situation dans les courts et moyens courriers. INRS. (2007); Documents pour le Médecin du Travail (111): 307–333.

[19] Feijó D Luiz RR Camara VM. Common mental disorders among civil aviation pilots. Aviat Space Environ Med. (2012) 83:509–13. doi: 10.3357/ASEM.3185.2012, PMID: 22606868

[20] Société Française d’Alcoologie – SFA. Mésusage de l’alcool dépistage, diagnostic et traitement Recommandation de bonne pratique. Alcoologie et Addictologie. (2015) 37:5–84.

[21] Guelfi JD Rouillon F Mallet L. Manuel de psychiatrie. 4e éd ed. Issy-les-Moulineaux: Elsevier Masson (2021).

[22] Flight Standards Division, Airworthiness division aviation safety and security department, Japan civil aviation bureau, Ministry of Land, infrastructure, Transport and Tourism. Guidelines for implementation of alcohol testing for flight crew etc. (2019). Available at: https://www.mlit.go.jp/koku/15_hf_000035.html

[23] Meerkerk G-J Njoo KH Bongers IMB Trienekens P Van Oers JAM. Comparing the diagnostic accuracy of carbohydrate-deficient transferrin, γ-Glutamyltransferase, and mean cell volume in a general practice population. Alcohol Clin Exp Res. (1999) 23:1052–9. doi: 10.1111/j.1530-0277.1999.tb04224.x, PMID: 10397290

[24] Karschner EL Schwilke EW Lowe RH Darwin WD Herning RI Cadet JL. Implications of plasma 9-tetrahydrocannabinol, 11-Hydroxy-THC, and 11-nor-9-Carboxy-THC concentrations in chronic Cannabis smokers. J Anal Toxicol. (2009) 33:469–77. doi: 10.1093/jat/33.8.469, PMID: 19874654 PMC3159863

[25] Katz G Durst R Zislin Y Barel Y Knobler HY. Psychiatric aspects of jet lag: review and hypothesis. Med Hypotheses. (2001) 56:20–3. doi: 10.1054/mehy.2000.1094 PMID: 11133250

[26] Nakata A Haratani T Takahashi M Kawakami N Arito H Kobayashi F. Association of Sickness Absence with poor sleep and depressive symptoms in shift workers. Chronobiol Int. (2004) 21:899–912. doi: 10.1081/CBI-200038104, PMID: 15646237

[27] American Psychiatric Association. American Psychiatric Association, éditeurs. Diagnostic and statistical manual of mental disorders: DSM-5. 5th ed. Washington, D.C.: American Psychiatric Association (2013). 947 p.

[28] Royant-Parola S. Insomnie et dépression. Annales Médico-psychologiques, revue psychiatrique. (2012) (cité 26 mars 2023); 170(3): 198–201. Available online at: https://linkinghub.elsevier.com/retrieve/pii/S0003448712000777

[29] Haute Autorité de Santé. Prise en charge du patient adulte se plaignant d’insomnie en médecine générale. Saint-Denis La Plaine: HAS. (2006). Available at: https://www.has-sante.fr/jcms/c_522637/fr/prise-en-charge-du-patient-adulte-se-plaignant-d-insomnie-en-medecine-generale

[30] Foyle MF Beer MD Watson JP. Expatriate mental health. Acta Psychiatr Scand. (1998) 97:278–83. doi: 10.1111/j.1600-0447.1998.tb10000.x PMID: 9570488

